# Facile Isolation of Adsorbent-Free Long and Highly-Pure Chirality-Selected Semiconducting Single-Walled Carbon Nanotubes Using A Hydrogen-bonding Supramolecular Polymer

**DOI:** 10.1038/srep18066

**Published:** 2015-12-14

**Authors:** Fumiyuki Toshimitsu, Naotoshi Nakashima

**Affiliations:** 1 Department of Applied Chemistry, Kyushu University, 744 Motooka, Nishi-ku, Fukuoka, 819-0395, Japan; 2 World Premier International Research Center Initiative-International Institute for Carbon-Neutral Energy Research (WPI-I^2^CNER), Kyushu University; 3 JST-CREST, 5 Sanbancho, Chiyoda-ku, Tokyo 102-0075, Japan

## Abstract

The ideal form of semiconducting-single-walled carbon nanotubes (sem-SWNTs) for science and technology is long, defect-free, chirality pure and chemically pure isolated narrow diameter tubes. While various techniques to solubilize and purify sem-SWNTs have been developed, many of them targeted only the chiral- or chemically-purity while sacrificing the sem-SWNT intrinsic structural identities by applying strong ultra-sonication and/or chemical modifications. Toward the ultimate purification of the sem-SWNTs, here we report a mild-conditioned extraction of the sem-SWNTs using removable supramolecular hydrogen-bonding polymers (**HBP**s) that are composed of dicarboxylic- or diaminopyridyl-fluorenes with ~70%-(8,6)SWNT selective extraction. Replacing conventional strong sonication techniques by a simple shaking using **HPB**s was found to provide long sem-SWNTs (>2.0 μm) with a very high D/G ratio, which was determined by atomic force microscopy observations. The **HBP**s were readily removed from the nanotube surfaces by an outer stimulus, such as a change in the solvent polarities, to provide chemically pure (8,6)-enriched sem-SWNTs. We also describe molecular mechanics calculations to propose possible structures for the **HBP**-wrapped sem-SWNTs, furthermore, the mechanism of the chiral selectivity for the sorted sem-SWNTs is well explained by the relationship between the molecular surface area and mass of the **HBP**/SWNT composites.

SWNTs have attracted special interests as materials with remarkable electronic, mechanical, thermal and photophysical properties due to their unique one-dimensional structures^1^,^2^,^3^,^4^. Their characteristic quantum-confined structures due to their chiral indices (n,m) provide unique opto-nanoelectronic behaviors^5^,^6^,^7^,^8^,^9^. Therefore, the separation/purification of the sem-SWNTs based on their chiralities is one of the most important issues in the science and applications of carbon nanotubes^10^,^11^. However, to achieve chiral- and chemical-purifications at the same time is still challenging because most of the commercially supplied as-produced SWNTs contains both sem-SWNTs and metallic-SWNTs as well as metal catalysts and other carbon forms. A typical purification requires solution-phase processes using surfactants^12^, which also chemically contaminate the sem-SWNTs by changing the surrounding environments that alter the intrinsic properties of the sem-SWNTs^13^,^14^,^15^.

Many attempts toward the goal of selective sem-SWNT sorting have been reported and the typical methods include polymer-wrapping^16^,^17^,^18^,^19^,^20^,^21^,^22^,^23^,^24^, density gradient ultracentrifugation^25^,^26^ and gel chromatography^27^,^28^,^29^. Such methods are powerful for the selective chirality sorting; however, complete removal of the used adsorbents from the sorted SWNT surfaces is difficult^30^,^31^. Even after the suitable adsorbent removal procedures, adsorbent molecules still remain on the sorted SWNTs^32^,^33^,^34^,^35^. We previously reported an efficient SWNT sorting using a supramolecular coordination polymers (**CP**s)^36^ followed by complete removal of the **CP**s based on dynamic supramolecular coordination chemistry^37^, which was the first report to utilize a supramolecular system for the selective sorting of the sem-SWNTs not containing the used adsorbent. In contrast, other reported polymers for the chemical purification of SWNTs usually lack a chirality sorting ability and/or adsorbent-removal process^30^,^31^,^32^,^33^,^37^,^38^. Furthermore, in order to maintain the original sem-SWNT properties after the sorting processes, structural identities of the sem-SWNTs, such as the length and crystallinity of the graphitic surface structure, must be preserved^39^,^40^, which is very difficult under conventional solubilizing/sorting experimental conditions using strong sonication^25^,^41^,^42^,^43^, which is a destructive process for the SWNTs. This issue is especially important for the sem-SWNTs with smaller diameters, such as chemical vapor deposition-produced HiPco- and CoMoCAT-SWNTs having much larger band gaps than those of the SWNTs with large diameters synthesized by the arc-discharged or laser-ablation method^44^. Such small diameter SWNTs are promising materials for use in optoelectronic device applications, such as thin film transistors, transistors, sensors, etc.

Here, we report a mild and highly chirality-selective extracting method for the chemically pure smaller diameter sem-SWNT sorting using newly-designed and synthesized supramolecular hydrogen-bond polymers (**HBP**s) as shown in Fig. 1 together with the concept of this study. The sem-SWNT selectivity is programmed in the **HBP**s by introducing fluorene moieties, which features molecular recognition of the sem-SWNTs^16^,^17^,^18^,^20^,^21^,^22^,^34^,^35^,^36^, and the formation of a linear polymer conformation achieved by the combination of a carboxylic acid and 4-aminopyridine on both ends of the fluorene is designed for the selective sorting of smaller diameter SWNTs. As a **HBP**, we designed and synthesized two building blocks, 2,7-bis-4-aminopyridyl-9,9'-dioctylfluorene (**1**) and 2,7-dicarboxyl-9,9'-dioctylfluorene (**2**). Furthermore, the simplicity of the **HBP** conformations aided in determining the mechanism of the chiral-selectivity of the **HBP** by considering the molecular surface area and the molecular weight of the composite with sem-SWNTs, which is the first trial to evaluate the selectivity extracting specific chiral sem-SWNTs based on the dynamic supramolecular chemistry. In this study, our goal is to extract long sem-SWNTs with smaller tube diameters while retaining their structural properties. During this study, using a concept that is similar to our previous report based on dynamic supramolecular chemistry^36^, Pochorovski *et al.*^44^ focused on the extraction of large-diameter SWNTs and reported the extraction of arc-discharge-produced SWNTs with diameters in the range of 1.28–1.39 nm using an **HBP** with a wide zigzag polymer structure as well as the removal of the used polymer adsorbent. The dispersion of such larger-diameter tubes using the reported **HBP** with sheet- or ribbon-structures needed sonication by a tip-sonicator with a higher-power^38^,^44^. The SWNTs with small tube diameters have highly tensioned graphitic surfaces, and for the dispersion of such tubes, sheet-shaped solubilizers and high power sonication are not suitable. Instead, our designed and synthesized linear **HBP** dispersed the SWNTs with small diameters under mild condition, which is one of advantages of this study.

## Results and Discussion

### Formation of an HBP from compounds 1 and 2

It has been reported that the selective extraction of the sem-SWNTs using fluorene-based copolymers is only possible in toluene and related aromatic solvents^16^,^20^,^22^; however, the synthesized compound **2** was insoluble in such solvents, but very soluble in polar solvents such as acetone. Since the complementary hydrogen-bonding of a carboxylic acid and 2-aminopyridine is a competitive reaction over self-dimerization by themselves^45^, ^46^, ^47^,^45^, ^46^, ^47^, we carefully chose the solvent combination. In this study, we used a mixed solvent of toluene and acetone. The complementary **HBP** formation in this mixed solvent was confirmed based on the ^1^H NMR titration, in which we recognized a shift in the aromatic region of both building blocks, **1** and **2**, and an indicative shift of the amino-proton of **1** from 5.0 to 5.2 ppm as shown by the arrows in Supplementary Fig. 1 (for the entire NMR spectra, see Supplementary Fig. 2). Furthermore, no end-capping molecule was observed, indicating that the **HBP** has a high molecular weight^38^,^44^.

### Solubilization of HiPco-SWNTs using the HBP

**Figure 2:**
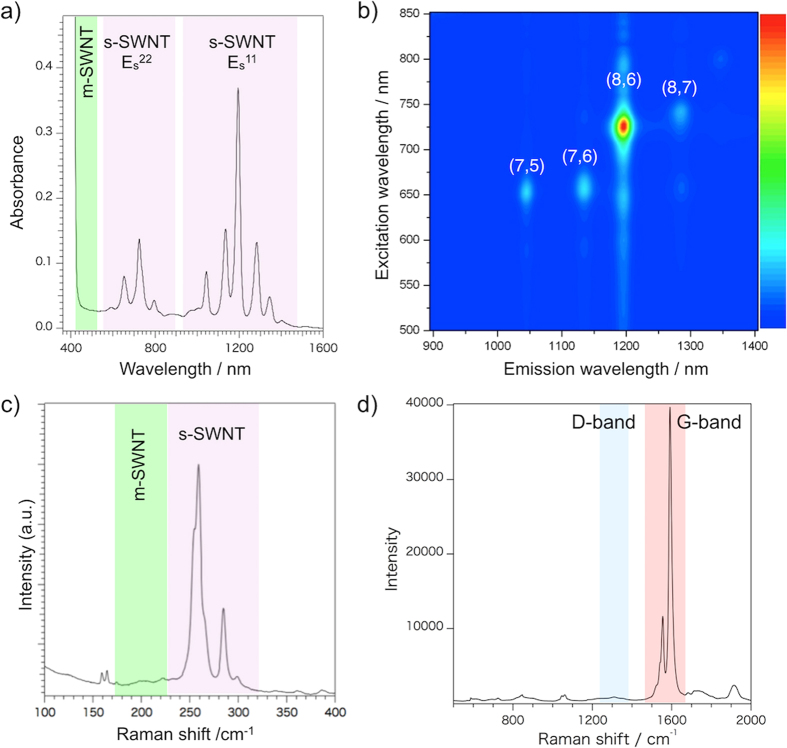
Vis-NIR Absorption, PLE and Raman spectra of sorted sem-SWNTs. (**a**) Vis-NIR absorption spectrum and (**b**) PLE mapping of the sorted sem-SWNTs (optical path length = 1.0 cm) using a mixture of compounds **1** (1.0 mM) and **2** (1.0 mM) in toluene/acetone. Raman spectra (excitation with a 633-nm laser) of (**c**) 100 ~ 400 cm^−1^ (RBM) and (**d**) 500 ~ 2000 cm^−1^ (D/G) regions of the sorted sem-SWNTs.

We first confirmed that both **HBP**-building blocks, **1** and **2,** have no ability to dissolve the SWNTs by themselves (Supplementary Fig. 3). The method for the solubilization of the smaller-diameter SWNTs using the **HBP** formed by the hydrogen binding of **1** and **2** is quite different from conventional techniques^20^,^21^,^22^,^23^,^24^,^25^,^31^,^33^,^34^,^35^,^36^,^37^, in which 1 h or longer sonication time is required. Under such severe experimental conditions, the **HBP** was found to be unable to retain their hydrogen bonding structure and did not solubilize the SWNTs at all (Supplementary Fig. 4). Hence, in this study, instead of heavy-duty sonication, an ~10-min mild sonication with a bath-type sonicator followed by an ~1-week shaking using a shaker were done to solubilize the as-produced HiPco-SWNTs. Figure 2a shows the Vis-NIR absorption spectra of the solubilized SWNTs collected by centrifugation at 10,000 × *g*, in which sharp bands corresponding to the Es^11^ (1000–1600 nm) and the Es^22^ (600–800 nm) of the sem-SWNTs are clearly observed, while no such absorption peak was detected in the metallic-SWNT region (400–600 nm). This behavior indicates that the **HBP** formed by **1** and **2** exclusively solubilized and extracted the sem-SWNTs, which is similar to the results using the other fluorene (co)polymers^20^,^21^,^22^,^34^,^35^,^36^. Furthermore, only four major absorption peaks were observed in both the Es^11^ and Es^22^ regions, that are ascribable to the chiralities of the sem-SWNTs with (n,m) = (7,5), (7,6), (8,6) and (8,7). Compared to the other fluorene-based polymers, **HBP** in this study has a high chiral selectivity. The estimated yield of extraction was ~9% in weight, which is comparable to that using PFOs. Related to extraction efficiency, we like to emphasize that our **HBP** can be reused, because it detaches from the SWNT surfaces after the selective extraction, which contributes to higher extraction efficiency (see the Supplementary Fig. 6).

In order to determine the abundance according to the chirality of the sorted sem-SWNTs, photoluminescense vs. excitation (PLE) mapping^6^ was measured (Fig. 2b); the result is summarized in Supplementary Table 1. The amount ratios of the extracted sem-SWNT chiralities of (7,5), (7,6), (8,6) and (8,7) were 7, 15, 71 and 7%, respectively. Compared to many conventional fluorene-based copolymers^20^,^21^,^22^,^34^,^35^, the **HBP** was found to be highly selective in sorting the (8,6)-sem-SWNTs from the as-prepared HiPco-SWNTs. We will address this chirality recognition behavior based on molecular mechanics simulations later.

In order to evaluate the chiral-purity and the degree of the SWNT crystalline structure of the extracted sem-SWNTs, the Raman spectra excited at 633-nm were measured, and the results shown in Fig. 2c, in which sem-SWNT peaks (240–300 cm^−1^) in the radial breathing mode (RBM) region were observed, while no metallic peaks around 200–240 cm^−1^ were detected. Furthermore, as shown in Fig. 2d, the intensity of the defect band (D-band) of the sorted SWNTs around 1310 cm^−1^ was much lower than that of the G-band; thus the D/G ratio was determined to be ~1/80, which is remarkably higher than those of the as-prepared SWNTs since even in commercially available very high quality HiPco-SWNTs, the D/G ratios are ~1/6. *Such a very high crystalline SWNT sorting is one of the advantages of this study over previous studies*.

**Figure 3:**
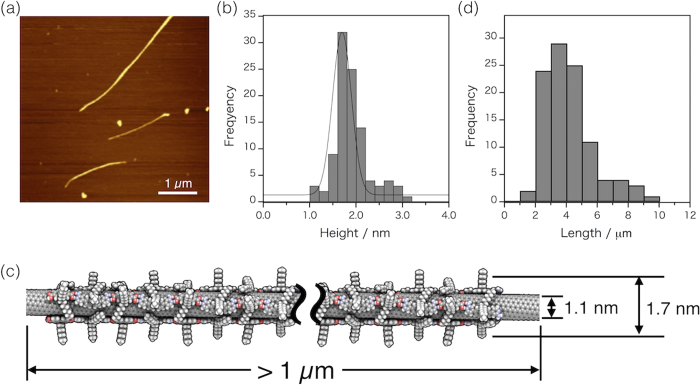
(**a**) A representative AFM image of isolated sem-SWNTs wrapped by **HBP** and (**b**) height distribution histogram. (**c**) A schematic illustration of the estimated dimensions for the structure-optimized composite structure of (8,6)sem-SWNT wrapped by **HBP**. (**d**) Length distribution histogram obtained from ten AFM images of sorted sem-SWNTs.

Atomic force microscopy (AFM) measurements also revealed the advantage of this study using the mild-conditioned-extraction procedure. The observed average height from the AFM image (Fig. 3a,b) is 1.21 ± 0.04 nm, which well agrees with the value calculated from the simulated individualized structure of the **HBP**-wrapped (8,6) SWNT (Fig. 3c). In the AFM image, we observed very long tubes (~10 μm) and the average length of one hundred of the individualized SWNTs reaches ~3.5 μm (Fig. 3d), which is much longer than those of the SWNTs based on the conventional sonication technique, in which the average diameter is usually less than 1.5 μm when using HiPco-SWNTs as the material. *The obtained long tube sorting is due to the very weak dispersion method using a shaker, which is one more advantage of this study*.

### Molecular mechanics simulations on the composite of SWNTs and HBPs

**Figure 4:**
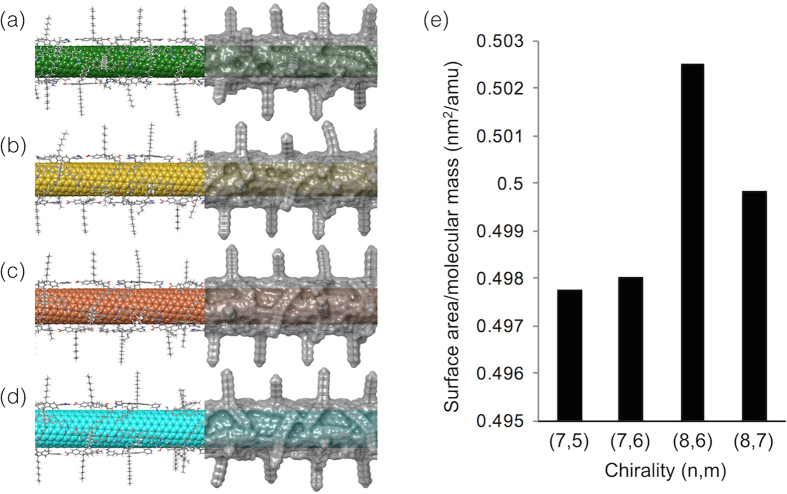
Optimized structures of HBP-wrapped sem-SWNTs. Molecular structures (left half) and molecular surfaces (right half) of **HBP**-wrapped (**a**) (7,5), (**b**) (7,6), (**c**) (8,6) and (**d**) (8,7) sem-SWNTs. (**e**) Molecular surface area and molecular mass for the **HBP**-wrapped four sem-SWNTs ((7,5), (7,6), (8,6) and (8,7), respectively). (**f**) Quotient values of molar mass divided by the surface area of the **HBP**-wrapped four sem-SWNTs shown with the chirality of sem-SWNTs.

As already described, the **HBP** showed a high chiral-selectivity for the sem-SWNTs compared to the other previously reported (co)polymers. For a deeper understanding of this behavior, especially for the interaction of the **HBP** and the sem-(8,6) SWNTs, the optimized conformations of the **HBP**s on the tubes were modeled using molecular mechanics simulations (Fig. 4a–d). In order to rationally wrap the SWNT surfaces with the **HBP**s, four strips of the **HBP** chains were placed on a 20-nm long sem-SWNT. After the structure optimization, all the included hydrogen-bonds between the molecules, **1** and **2**, maintained the rational distance of 1.98~2.12 Å in all the chiralities. The resulting conformations of the **HBP** were close to a linear structure, which reflected the programmed strict bond-angle and bond-length nature of the hydrogen bonds. Thus, the **HBP** wrapping is not very flexible compared to the helical wrapping manners presented by many other fluorene copolymers composed of a covalent- or coordination bonding^20^,^21^,^22^,^34^,^35^,^36^, which lead to a weak chirality selectivity.

In an effort to elucidate the specific chiral-selectivity of the **HBP** on the sem-SWNTs, we examined the relationship between the ratios of the experimentally-sorted SWNT chiralities and the theoretically obtained structural information of the composites of the SWNT and **HBP**. Gomulya *et al.*^48^ estimated the interactions between several fluorene oligomers and two chiralities of SWNT by the surface area and the binding energy using a molecular dynamics simulation. In our study, by contrast, we used only one kind of polymer (**HBP)** and it will not give a comparable binding energy difference. The relationship between the surface area and molecular weight of the composites was then discussed. Noteworthy is that the accurate determination of the molar density for such hollow and open-end SWNTs with an encapsulation ability is very difficult, thus we regard the surface area and molecular weight as an indicator of the stabilization of the **HBP**-wrapped SWNTs in solvents. As shown in Supplementary Table 2, the molecular surface area and molecular mass naturally increased with the increase in the number of atoms in the given composites, while the values of the molar mass divided by the surface area show a local maximum for the composite of (8,6)-SWNT (Fig. 4e). This behavior reflects the amount of the **HBP**’s molecular surface area and uncovered area of the SWNT surfaces, which strongly influences the stability and the solubility of the composites. As a result, the composite of the sem-(8,6)SWNT and **HBP**s showed the highest stability in the calculation analysis that strongly supports the specific recognition behavior of the **HBP**. Meanwhile, a conventional analysis using stabilizing energy did not provide a clear difference as shown in Supplementary Table 3. Thus, our approach to theoretically estimate the chirality selectivity by means of the molecular surface area and the molecular weight is a good method to predict the interactions and stabilization of the SWNT composites in the solution phase.

### Removing HBPs from sem-SWNT

**Figure 5:**
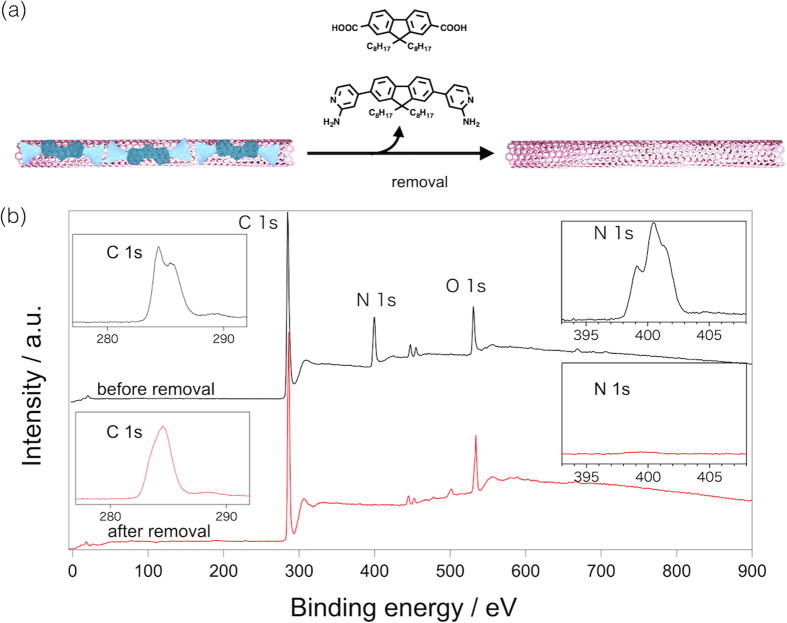
Adsorbent removal from sem-SWNTs. (**a**) Schematic drawing of the removal of **HBP** from the extracted sem-SWNT and (**b**) XPS spectra of the sem-SWNTs before (black line) and after (red line) the removal of **1** and **2** with the magnified C1s and N1s region (the insets).

Removal of the **HBP** from the sem-SWNT surfaces is very important. As already described, compounds **1** and **2** were unable to form hydrogen bonding in many solvents except for the 1:1 toluene/acetone mix solvent. The removal of the **HBP** polymer was quite easy; namely, we completely removed the **HBP** by a very simple intense washing with a good solvent and obtained chemically pure sem-SWNTs. Typically, the **HBP**-wrapped sem-SWNTs solution (3 ml) was added to excess acetone (50 ml), then sonicated for 10 min to produce a black precipitate, which was filtered, then thoroughly washed with acetone to provide a black solid. The product was analyzed by the X-ray photoelectron spectroscopy (XPS)^36^ and the result is shown in Fig. 5, in which we observed that the carbon peaks at around 284 eV became sharper after the removal-treatment, indicating the disappearance of the *sp*^3^ alkyl carbon derived from the **1** and **2** monomers. A similar behavior was observed in the nitrogen and oxygen regions; namely, no nitrogen peak was detected after the removal-treatment. We observed a peak in the oxygen region after the removal, which is assigned not to the adsorbent, but the adsorbed water since a peak appeared at 533 eV. Noteworthy to recognize is that compounds **1** and **2** were not damaged during the removal process, hence they are readily reusable, indicating a highly efficient SWNT sorting in this study. *This easy adsorbent removal is one more advantage of this study.*

The recovered compounds **1** and **2** were identified by ^1^H NMR spectroscopy (Supplementary Fig. 5). The complete recovery was confirmed by measuring the weight of the recovered compounds. The recovered **1** and **2** were reused to solubilize the as-produced SWNTs, and the obtained result is shown in Supplementary Fig. 6, which is almost identical to Fig. 2a.

## Conclusions

In conclusion, a method for the efficient extraction of highly pure long-length sem-SWNTs with minute defects has been achieved using the hydrogen bonding polymer **HBP** along with a very mild conditioned-solubilizing/extracting procedure based on dynamic supramolecular chemistry. The amount ratios of the extracted sem-SWNTs with chiralities of (n,m) = (7,5), (7,6), (8,6) and (8,7) were 7, 15, 71 and 7%, respectively. A facile and highly selective one-pot sem-(8,6)SWNT extraction is of interest, and the behavior was explained by molecular mechanics simulations.

The summarized advantages of this study using a very easy handling technique are: i) *very high crystalline sem-SWNT sorting*, ii) selective (8,6)SWNT sorting, iii) long sem-SWNT sorting and iv) an easy and complete removal of the adsorbent from the sorted sem-SWNT surfaces. Such an efficient and easy extraction of the sem-SWNTs is a great advantage in the science of the chirality selective SWNT separation. The study is highly important since the present sorted sem-SWNTs are highly pure (adsorbent free), chiral selective and sufficiently-long, which satisfy the strong demand in the use of such materials in many fundamental research studies and applications.

## Methods

### Materials

Compounds **1** were synthesized by the Suzuki-Miyaura coupling between 2,7-dibromo-9,9'-dioctylfluorene and 2-amino-4-iodopyridine (Supplementary Fig. 6) and **2** were prepared according to the literature^48^. HiPco-SWNTs were purchased from Unydim (lot# P0261) and used as received. Toluene and acetone were purchased from Tokyo Chemical Industry Co., Ltd. Japan (spectra analysis grade).

### Solubilization of SWNTs using HBP

Into a 13.5 mL vial, 1.72 mg (3.00 mmol) of **1** and 1.44 mg (3.00 mmol) of **2** were solubilized using 1.5 mL of acetone and 1.5 mL of toluene, then 1.0 mg of the as-produced HiPco-SWNT were added. A brief 10-min sonication using a bath-type sonicator (5510 J-MTH, Emerson Japan, Ltd., Bransonic) was applied to roughly debundle the SWNTs followed by shaking (ASONE Corporation, Neo Shaker, 700 rpm) for ~1w. After a 1 h centrifugation, the resulting supernatant was corrected and analyzed (for details, see Supplementary Information).

### Molecular mechanics simulations

The molecular-mechanics simulations were carried out using the MacroModel program (Schrödinger, version 9.8) with the OPLS-2005 force field. The dielectric constant of toluene (2.3) was used in the calculations. Minimization of the calculations was carried out by using the Polak-Ribiere conjugate gradient (PRCG) with a convergence threshold on the gradient of 0.05 kJ/mol. Default values have been used for all the other parameters. The molecular surface was calculated for the optimized structures of the composite between the sem-SWNTs and HBP with probe diameter of 0.1 nm and van-der-Waals radius scale was 1.0.

### Removal of HBP from sorted sem-SWNT surface

The sem-SWNT solutions with **HBP** were filtered using PTFE filters (AVANTEC, 0.1 μm pore size). The collected black solids were dispersed in 50 ml of acetone and sonicated for 10 min. to immediately generate a black suspension, which was filtered through a PTFE filter (AVANTEC, 0.1 μm pore size) followed by three washings with 10 ml of acetone and confirming no absorption of **1** and **2** observed in the UV-vis absorption spectrum of the filtrates. The obtained SWNT solid was dried in vacuo and used for the XPS analysis.

## Additional Information

**How to cite this article**: Toshimitsu, F. and Nakashima, N. Facile Isolation of Adsorbent-Free Long and Highly-Pure Chirality-Selected Semiconducting Single-Walled Carbon Nanotubes Using A Hydrogen-bonding Supramolecular Polymer. *Sci. Rep.* **5**, 18066; doi: 10.1038/srep18066 (2015).

## Supplementary Material

Supplementary Information

## Figures and Tables

**Figure 1:**
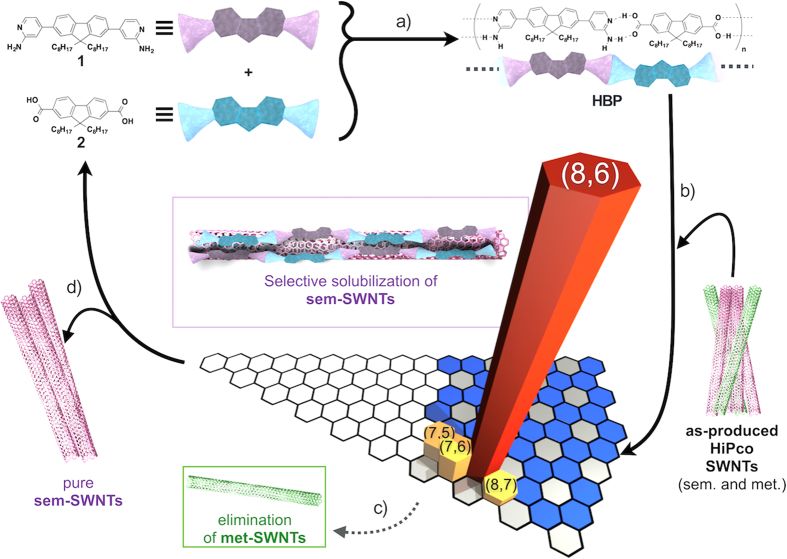
Schematic illustration of the purification cycle of sem-SWNTs using HBP made of 1 and 2. (**a**) Chemical structures of **1**, **2** and **HBP** formed by **1** and **2**. (**b**) Solubilization/sorting of the sem-SWNTs using the **HBP** takes place with (**c**) the elimination of met-SWNTs in toluene-acetone mixed solvent. (**d**) The reversible formation and deformation of **HBP** enabled regeneration of fresh **1** and **2** after the separation of chemically-pure sem-SWNTs.
