# Supplementary material for: Facile Isolation of Adsorbent-Free Long and Highly-Pure Chirality-Selected Semiconducting Single-Walled Carbon Nanotubes Using A Hydrogen-bonding Supramolecular Polymer
Source: Sci Rep. 2015 Dec 14;5:18066. doi: 10.1038/srep18066 (PMC4677298; doi:10.1038/srep18066)
Supplement: Supplementary Information [file srep18066-s1.pdf]

Supplementary Information

for

Facile Isolation of Adsorbent-Free Long and Highly-Pure Chirality-Selected Semiconducting Single-Walled Carbon Nanotubes Using A Hydrogen-bonding Supramolecular Polymer

Fumiyuki Toshimitsu[a] and Naotoshi Nakashima*[a,b,c]

[a] Department of Applied Chemistry, Kyushu University, 744 Motooka, Nishi-ku, Fukuoka, 819-0395, Japan

[b] World Premier International Research Center Initiative-International Institute for Carbon-Neutral Energy Research (WPI-I^{2}CNER), Kyushu University

[c] JST-CREST, 5 Sanbancho, Chiyoda-ku, Tokyo 102-0075, Japan

*e-mail: nakashima-tcm@mail.cstm.kyushu-u.ac.jp

Instruments

^1H NMR spectra, high-resolution mass spectra (HRMS), UV/Vis-NIR absorption spectra, PL spectroscopic analysis, 2D PL mapping and Raman spectra (excitation at 633-nm) were recorded using a Bruker AV300M spectrometer, a Bruker MicroTOF-QIII electrospray ionization time-of-flight (ESI-TOF) mass spectrometer, a V-670 (Jasco), a Horiba Jobin Yvon spectrofluorometer (FluorologR-3 with FluorEssence) and a RAMANtouch spectrometer (Nanophoton Corporation), respectively. The AFM images were recorded by an Agilent Technologies Agilent 5500 and freshly cleaved mica was used as the substrate. The X-ray photoelectron spectroscopy (XPS) spectra were measured using an AXIS-ULTRA^{DLD} (Shimadzu). The molecular-mechanics simulations were carried out using the MacroModel program (Schrodinger, version 9.8) with the OPLS-2005 force field. The dielectric constant of toluene (2.3) was used in the calculations. Minimization of the calculations was carried out by the Polak-Ribiere conjugate gradient (PRCG) with a convergence threshold on the gradient of 0.05 kJ/mol. Default values have been used for all the other parameters.

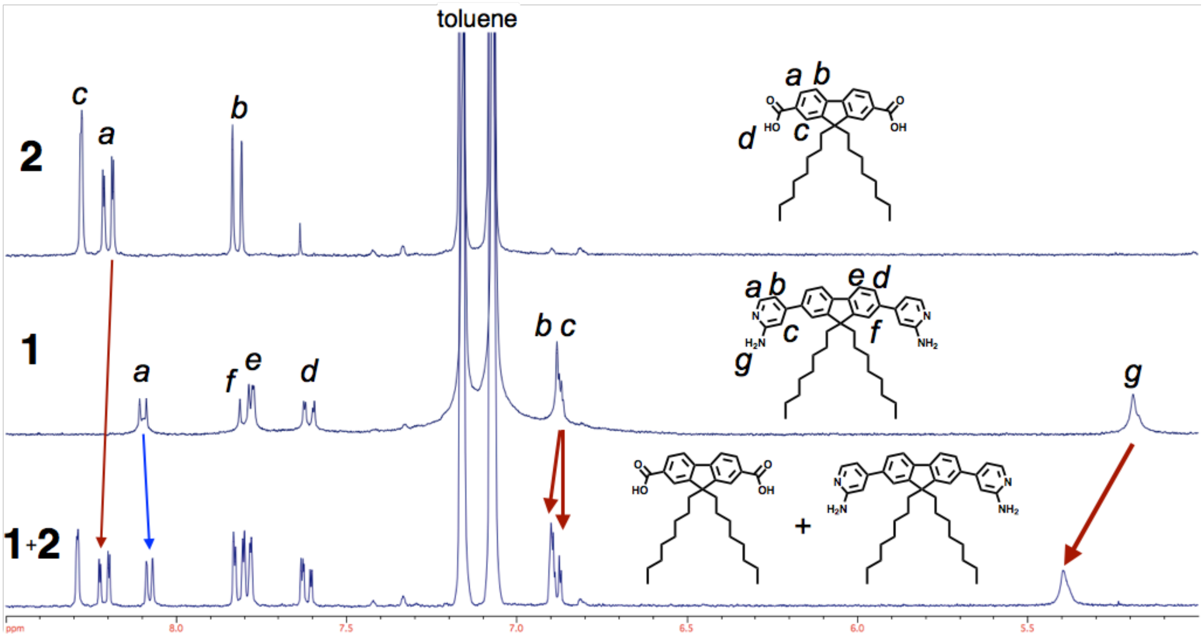

Supplementary Figure 1. [1]**H NMR spectra** (300 MHz, toluene-d_8: acetone-d_6=1:1, selected region) of compounds **1**, **2** and an equimolar mixture of **1** and **2**. The red arrows indicate the shift in the corresponding peaks. The concentration of each molecule in the mixed solution is 2.0 mM.

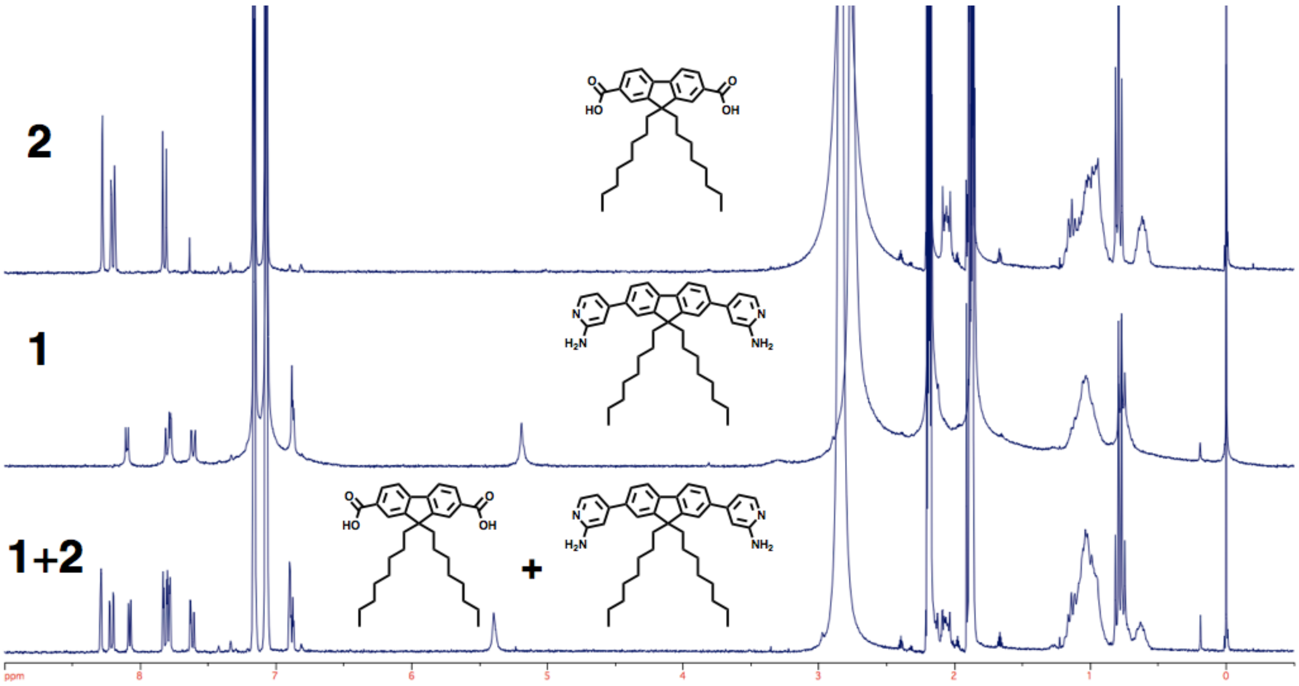

Supplementary Figure 2. **^{1}H NMR spectra** (300 MHz, toluene-d_8: acetone-d_6=1:1) of compounds **1**, **2** and an equimolar mixture of **1** and **2** (2.0 mM).

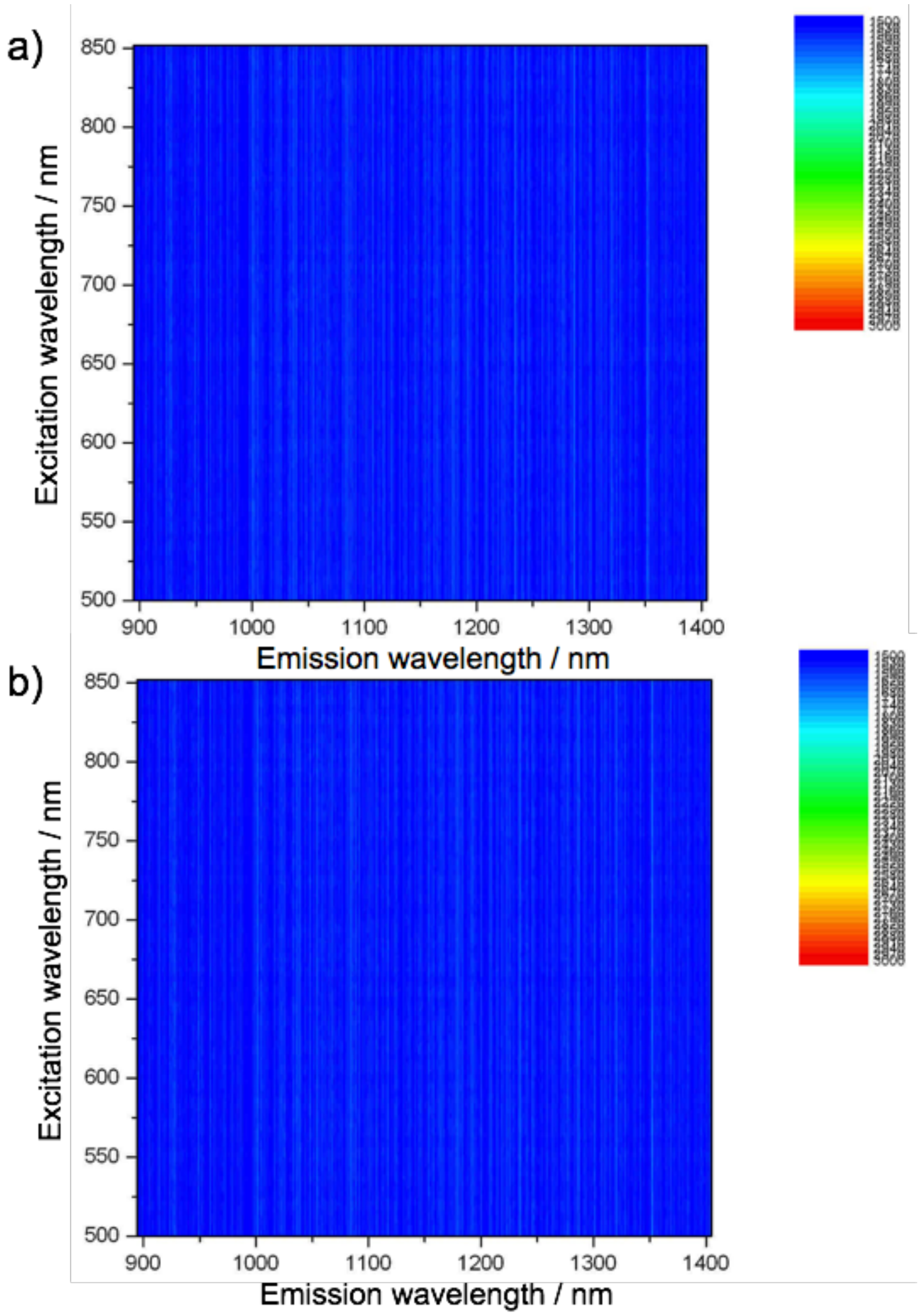

Supplementary Figure 3. **PLE mappings for the SWNT region** of a solution obtained by a shaking-based solubilization procedure using (a) **1** or (b) **2** in a mixed solvent of toluene and acetone (1/1 vol%). No peak in the SWNT absorption area was observed.

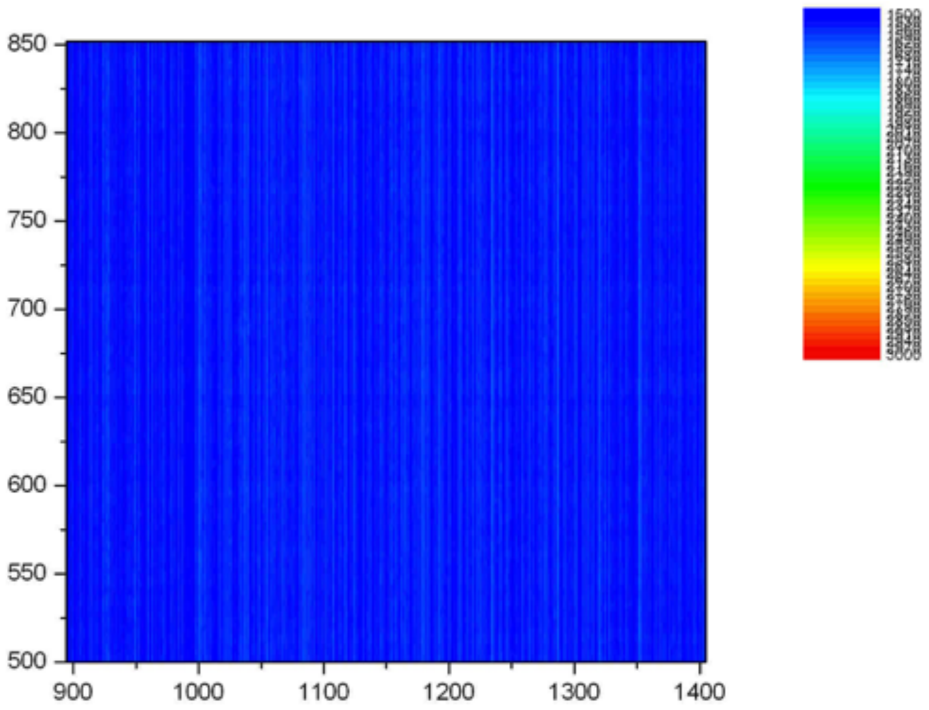

Supplementary Figure 4. **PLE mapping of a solution** obtained by a sonication-based solubilization procedure for the as-produced SWNTs using **1** and **2** in a mixed solvent of toluene and acetone (1/1 vol%). No peak in the SWNT absorption area was observed.

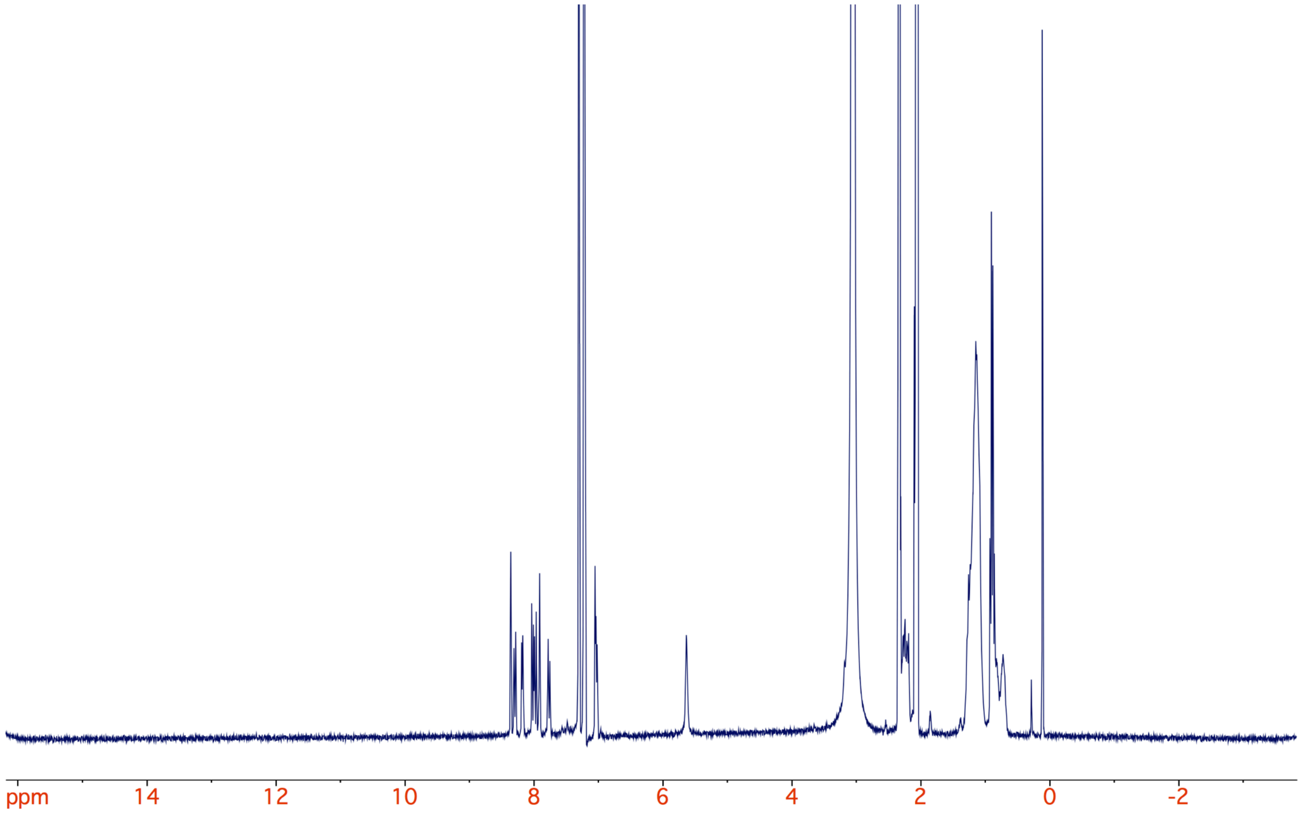

Supplementary Figure 5. **^{1}H NMR spectra** (300 MHz, toluene-d_8: acetone-d_6=1:1, selected region) of the recovered compounds **1**, **2** and an equimolar mixture of **1** and **2**.

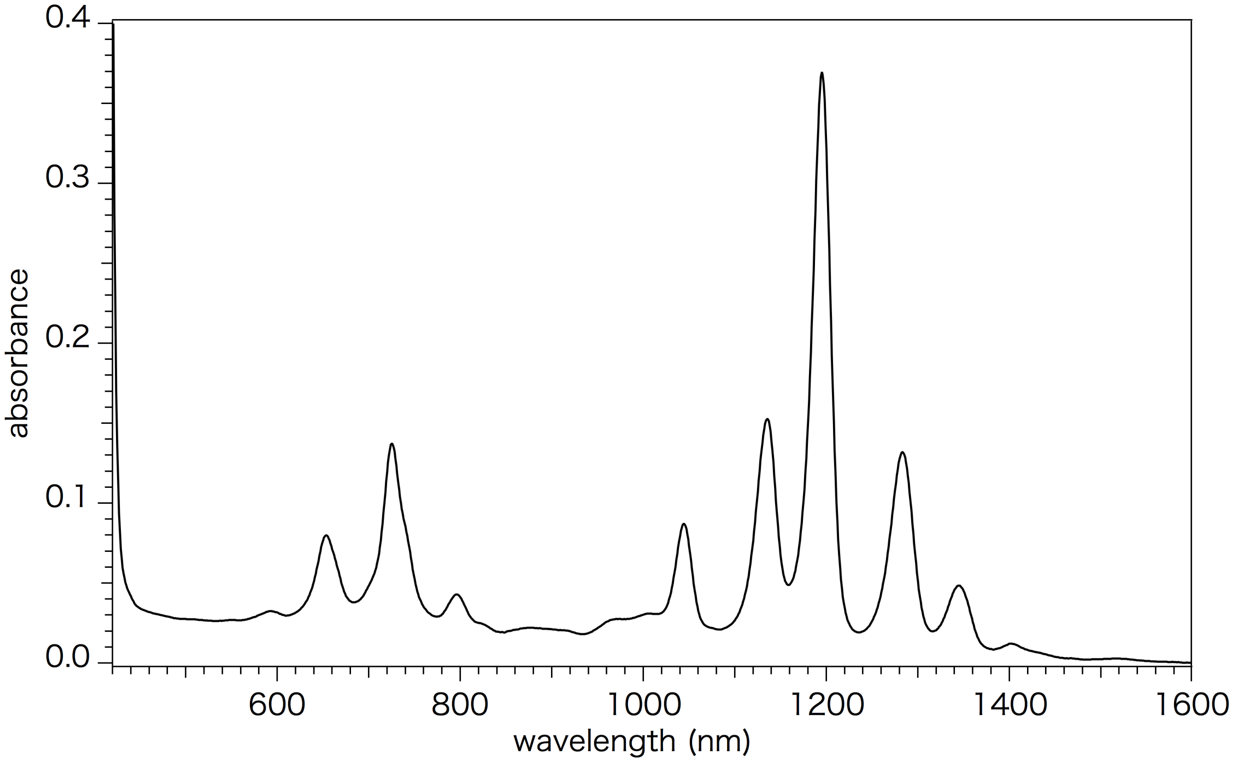

Supplementary Figure 6. **Vis-NIR Absorption spectrum of sorted sem-SWNTs using recovered HBP.** Measured using a mixture of compounds **1** (1.0 mM) and **2** (1.0 mM) in toluene/acetone (optical path length was 1.0 cm.).

Supplementary Table 1. **Calibrated contents of the SWNT species** deduced from the PLE mapping of the extracted sem-SWNTs using the **HBP**.

(n,m)	Emission peak shift	PL peak intensity	Calculated PL intensity	Calibrated PL peak intensity	Calibrated content
(7,5)	1043	830.28	0.71	589.50	7.1
(7,6)	1139	2610.88	0.47	1227.11	14.9
(8,6)	1186	12046.17	0.49	5902.62	71.5
(8,7)	1279	1796.23	0.30	538.87	6.5

Supplementary Table 2. The molecular surface area and the molecular mass calculated using the optimized structures of the composites of the sem-SWNTs with the **HBP**.

SWNT	Surface area of **HBP**-wrapped SWNT	Molecular mass of **HBP**-wrapped SWNT
(7,5)	16227.098	32599.36
(7,6)	16911.202	33955.36
(8,6)	17485.23	34795.36
(8,7)	17974.228	35959.36

Supplementary Table 3. Calculated potential and stabilizing energies between the (n,m)SWNTs with **HBP**. The energies are in kcal/mol.

SWNT	Potential energy of SWNT E_{SWNT}	Potential energy of **HBP** $E_{\mathbf{HBP}}$	Total potential energy of **HBP**-wrapped SWNT $E_{SWNT} + E_{\mathbf{HBP}}$	Structure-optimized potential energy of **HBP**-wrapped SWNT $E_{complex}$	Stabilizing energy $E_{stabization}$
(7,5)	97469.625	174.441	98167.389	93962.953	-4204.436
(7,6)	98891.578	174.441	99589.342	95283.492	-4305.85
(8,6)	101321.125	174.441	102018.889	97664.852	-4354.037
(8,7)	103584.281	174.441	104282.045	99885.383	-4396.662
